# Supplementary material for: Identification of photoperiod-induced specific miRNAs in the adrenal glands of Sunite sheep (Ovis aries)
Source: Front Vet Sci. 2022 Jul 22;9:888207. doi: 10.3389/fvets.2022.888207 (PMC9354845; doi:10.3389/fvets.2022.888207)
Supplement: Supplementary file 1 [file Data_Sheet_1.ZIP › Supplementary file/miRDeep2 file.zip!/miRDeep2 file/Know_Structure/LP42A2/oar-mir-133.pdf]

miRBase precursor : oar-mir-133
Total read count : 768
oar-miR-133 read count : 744
remaining reads : 24

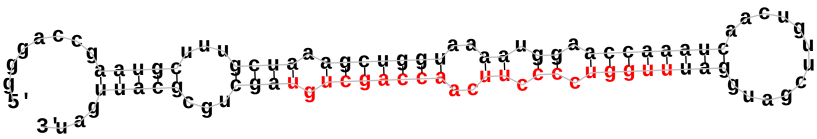

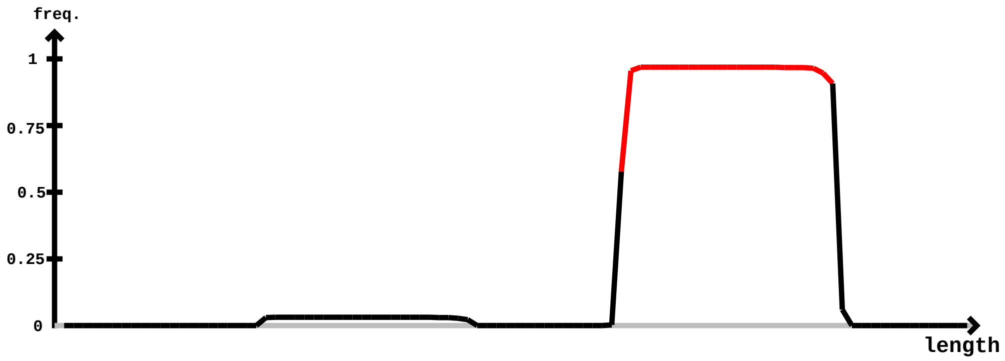


```
                                                                       oar-miR-133
5'- gggaccgaaugcuuugcuaaagcugguaaaauggaaccaaaucaacuguucgauggauuugguccccuucaaccagcuguagcugcgcauugau   -3'    exp
    .......(((((...((((.(((((((..((.((.((((((((............)))))))).)).))..))))))).))))...)))))...    reads      mm          sample
    ....................agcugguaaaauggaaccaa......................................................    2          0           seq
    ....................agcugguaaaauggaaccaaa.....................................................    4          0           seq
    ....................agcugguaaaauggaaccaaau....................................................    17         0           seq
    .....................gcugguaaaauggaacc........................................................    1          0           seq
    ........................................................auuugguccccuucaaccagcugu..............    2          0           seq
    .........................................................uuugguccccuucaacU....................    1          1           seq
    .........................................................uuugguccccuucaaccagc.................    1          0           seq
    .........................................................uuugguccccuucaaccagcu................    12         0           seq
    .........................................................uuugguccccuucaaccagcug...............    20         0           seq
    .........................................................uuugguccccuucaaccagcuA...............    3          1           seq
    .........................................................uuugguccccuucaaccagcugu..............    387        0           seq
    .........................................................uuugguccccuucaaccagcugua.............    18         0           seq
    ..........................................................uugguccccuucaaccagc.................    1          0           seq
    ..........................................................uugguccccuucaaccagcu................    2          0           seq
    ..........................................................uugguccccuucaaccagcug...............    6          0           seq
    ..........................................................uugguccccuucaaccagcuA...............    1          1           seq
    ..........................................................uugguccccuucaaccagcugu..............    252        0           seq
    ..........................................................uugguccccuucaaccagcugua.............    28         0           seq
    ...........................................................ugguccccuucaaccagcugu..............    10         0           seq
```
